# Supplementary material for: Parental Disease Specific Knowledge and Its Impact on Health-Related Quality of Life
Source: Children (Basel). 2022 Jan 11;9(1):98. doi: 10.3390/children9010098 (PMC8774947; doi:10.3390/children9010098)
Supplement: Supplementary file 1 [file children-09-00098-s001.zip › children-1482781-supplementary.pdf]

**Supplementary:** Parental disease specific training in pediatric hepatology

**Table S1 Questionnaire for parents/guardians**

**Current state of knowledge**
**With the following questions we would like to find out your current level of knowledge regarding your child's disease/transplantation. Our intention is not to test you, but to find out whether our current advice and information to you is sufficient. Therefore, we would like you to answer the questions honestly. Please, do not try to guess or research the answers. It does not matter if you do not know the answer to some of the questions.**

C1 Please, answer the following questions briefly and in bullet point form in your own words.

1. What is your child's underlying disease?

______________________________________________

______________________________________________

__________________

2. Please explain briefly what this disease entails.

______________________________________________

______________________________________________

______________________________________________

______________________________________________

______________________________________________

______________________________________

3.

| Which medications does your child currently take? | What are these medications for? | I do not know what this drug is given for |
|---|---|---|
| | | ☐ |
| | | ☐ |
| | | ☐ |
| | | ☐ |
| | | ☐ |

Which problems does your child currently that result from his/her liver disease?

______________________________________________

______________________________________________

______________________________________________

______________________________________________

4. What other health problems may your child have in the future due to the disease?

C2 Please tick the correct answers to the following questions. One or more answers may be correct. If you cannot answer a question or are not sure, please mark "I don't know".

1. Which functions of the liver do you know?

- ☐ Production of proteins and bile
- ☐ Metabolism of e.g. medication and alcohol
- ☐ Urine production
- ☐ Storage of sugar, fat and vitamins
- ☐ I do not know

2. What is the bile's function?

- ☐ It helps in the digestion of proteins
- ☐ It helps in the digestion of fats
- ☐ It helps in the digestion of sugars (carbohydrates)
- ☐ Without bile, the stool would be colorless
- ☐ I do not know

3. What are warning signs for biliary tract infection?

- ☐ Pale stool
- ☐ Fever
- ☐ Yellowing of the skin
- ☐ Vomiting
- ☐ I do not know

4. <u>What is abdominal dropsy?</u>

- ☐ Accumulation of fluid in the abdominal cavity
- ☐ It is fluids secreted from wounds, which may occur for a short time after transplantation
- ☐ It occurs when blood backs up in the portal vein
- ☐ It indicates a problem in the main artery
- ☐ I do not know

5. <u>What does the term “liver remodeling” mean?</u>

- ☐ The liver converts damaged tissue into healthy tissue
- ☐ The liver "learns" new functions
- ☐ The liver becomes increasingly scarred
- ☐ The transplanted liver adapts to the size of the recipient
- ☐ I do not know

6. <u>What does cholestasis mean?</u>

- ☐ Cholestasis means that bile stasis is present
- ☐ Cholestasis means that the liver is remodeling
- ☐ Cholestasis means that water occurs in the abdomen
- ☐ Cholestasis means having gallstones
- ☐ I do not know

7. <u>What is portal hypertension?</u>

- ☐ The blood in the portal vein flows into the liver without problems
- ☐ Blood in the portal vein backs up in front of the liver
- ☐ The blood in the portal vein flows with higher pressure
- ☐ Portal hypertension may result from liver remodeling

☐ I do not know

8. <u>What can happen in portal hypertension?</u>

☐ The spleen may swell

☐ Jaundice may occur

☐ Varicose veins may occur in the esophagus and stomach

☐ Abdominal dropsy may occur

☐ I do not know

9. <u>In case of which diseases in the immediate environment of the child (family, school, daycare center) you should report to the MHH?</u>

☐ Chickenpox

☐ diarrhea and vomiting

☐ Measles

☐ Hand-foot-and-mouth disease

☐ I do not know

10. <u>What are possible side of immunosuppressive drugs?</u>

☐ Increased growth of body hair

☐ swollen gums

☐ Arterial hypertension

☐ Kidney injury

☐ I do not know

11. <u>Which statements concerning the liver transplant waiting list are correct?</u>

☒ The children are registered at the Central Organ Transplant Center (Eurotransplant)

☐ Laboratory values (MELD score) must be sent to the central office on a regular basis

☐ The sex of the child (male/female) influences the score on the waiting list

☐ The blood group has an influence on the suitability of grafts

☐ I do not know

C3 For the following statements, please mark whether they are true or false. If you do not know an answer or are not sure, please tick "I do not know".

| | | True | false | I do not know |
|---|---|---|---|---|
| 1. | My child must reach a certain age to be able to get a new liver | ☐ | ☐ | ☐ |
| 2. | Children who have been on the transplant waiting list the longest get a new liver first | ☐ | ☐ | ☐ |
| 3. | The children who need a new liver most urgently get a new liver first | ☐ | ☐ | ☐ |
| 4. | It is possible that a child receives an organ offer although another child has more points on the waiting list | ☐ | ☐ | ☐ |
| 5. | My child should be as fully vaccinated as possible <u>before</u> transplantation | ☐ | ☐ | ☐ |
| 6. | Any vaccination may be performed immediately <u>after</u> transplantation | ☐ | ☐ | ☐ |
| 7. | Inactivated vaccines may be used from one year after transplantation | ☐ | ☐ | ☐ |
| 8. | The majority of organ donations are cadaveric donations from patients who have been diagnosed with brain death. | ☐ | ☐ | ☐ |
| 9. | There is a possibility that a relative donates a part of his/her liver | ☐ | ☐ | ☐ |
| 10. | The immunosuppressive drugs are needed to prevent the patient's own immune system from attacking the "new liver" | ☐ | ☐ | ☐ |
| 11. | The immunosuppressive drugs must be taken for the whole life | ☐ | ☐ | ☐ |
| 12. | The immunosuppressants should be taken during meals | ☐ | ☐ | ☐ |
| 13. | Immunosuppression leads to an increased susceptibility to infections | ☐ | ☐ | ☐ |
| 14. | The immunosuppressive drug trough level is important because it determines the dose that must be taken | ☐ | ☐ | ☐ |
| 15. | If the drug trough level is too high, there is a risk of rejection | ☐ | ☐ | ☐ |

| | | | |
|---|---|---|---|
| 16. My child is not allowed to eat before blood is drawn to determine the level of immunosuppressants | ☐ | ☐ | ☐ |
| 17. My child must not take his immunosuppressive drugs before blood is drawn to determine the drug trough level | ☐ | ☐ | ☐ |
| 18. In case of "rejection" the immune system reacts to the "new liver" | ☐ | ☐ | ☐ |
| 19. Rejection means that the transplanted organ is lost | ☐ | ☐ | ☐ |
| 20. Rejection may be related to too much immunosuppression | ☐ | ☐ | ☐ |
| 21. It is sufficient if my child goes for a medical checkup once a year | ☐ | ☐ | ☐ |
| 22. After transplantation, my child is entitled to a severely disabled person's card | ☐ | ☐ | ☐ |
| 23. My child must never do sports again after transplantation | ☐ | ☐ | ☐ |

D5 How would you rate <u>your level of knowledge</u> on the following topics:

| | Very good | Good | Sufficient | Little | Very Little |
|---|---|---|---|---|---|
| a) Illness of your child | ☐ | ☐ | ☐ | ☐ | ☐ |
| b) Anatomy of the liver (portal vein, bile ducts, etc.) | ☐ | ☐ | ☐ | ☐ | ☐ |
| c) Functions of the liver | ☐ | ☐ | ☐ | ☐ | ☐ |
| d) Technical terms such as liver remodeling, tarry stools, portal hypertension, etc. | ☐ | ☐ | ☐ | ☐ | ☐ |
| e) Proceedings during an inpatient stay | ☐ | ☐ | ☐ | ☐ | ☐ |
| f) Transplantation | ☐ | ☐ | ☐ | ☐ | ☐ |
| g) Proceedings before liver transplantation | ☐ | ☐ | ☐ | ☐ | ☐ |
| h) Proceedings during the in-patient stay for liver transplantation | ☐ | ☐ | ☐ | ☐ | ☐ |
| i) Proceedings after liver transplantation | ☐ | ☐ | ☐ | ☐ | ☐ |
| j) Meaning of laboratory values | ☐ | ☐ | ☐ | ☐ | ☐ |
| k) Medication of your child | ☐ | ☐ | ☐ | ☐ | ☐ |

| | | | | | | |
|---|---|---|---|---|---|---|
| l) | Immunosuppressants of your child | ☐ | ☐ | ☐ | ☐ | ☐ |
| m) | Diagnostic procedures, e.g. ultrasound, MRI, X-ray | ☐ | ☐ | ☐ | ☐ | ☐ |
| n) | Everyday life with the disease | ☐ | ☐ | ☐ | ☐ | ☐ |
| o) | Psychological support options | ☐ | ☐ | ☐ | ☐ | ☐ |
| p) | Possible assistance for the care of your child (e.g. care allowance, severely handicapped certificate, etc.) | ☐ | ☐ | ☐ | ☐ | ☐ |
| q) | Importance of nutrition | ☐ | ☐ | ☐ | ☐ | ☐ |

Table S2 The Ulm quality of life inventory for parents of children with a chronic illness ULQUI

B7 The following is about how you assess your current life situation. Please indicate to what extent the following statements apply to your behavior and state of mind over the past 7 days.[1]

| During the last 7 days | | Never | Rarely | Sometimes | Often | Always |
|---|---|---|---|---|---|---|
| a) | I was active and full of energy | ☐ | ☐ | ☐ | ☐ | ☐ |
| b) | I had physical complaints | ☐ | ☐ | ☐ | ☐ | ☐ |
| c) | I felt exhausted | ☐ | ☐ | ☐ | ☐ | ☐ |
| d) | I could sleep well | ☐ | ☐ | ☐ | ☐ | ☐ |
| e) | I could eat well | ☐ | ☐ | ☐ | ☐ | ☐ |
| f) | I felt fit | ☐ | ☐ | ☐ | ☐ | ☐ |
| g) | I had pain | ☐ | ☐ | ☐ | ☐ | ☐ |
| h) | I could concentrate well | ☐ | ☐ | ☐ | ☐ | ☐ |
| i) | I was worried | ☐ | ☐ | ☐ | ☐ | ☐ |
| j) | I had trouble to get up for something | ☐ | ☐ | ☐ | ☐ | ☐ |
| k) | I was hopeful and confident | ☐ | ☐ | ☐ | ☐ | ☐ |
| l) | I was resilient | ☐ | ☐ | ☐ | ☐ | ☐ |
| m) | I was irritable and nervous | ☐ | ☐ | ☐ | ☐ | ☐ |
| n) | I could realize my own wishes and needs | ☐ | ☐ | ☐ | ☐ | ☐ |
| o) | I was depressed and unhappy | ☐ | ☐ | ☐ | ☐ | ☐ |
| p) | I had plenty of opportunity to meet friends and acquaintances | ☐ | ☐ | ☐ | ☐ | ☐ |
| q) | I felt comfortable in the circle of my family | ☐ | ☐ | ☐ | ☐ | ☐ |
| r) | my relationship with my child(ren) was satisfactory | ☐ | ☐ | ☐ | ☐ | ☐ |

| | | | | | | |
|---|---|---|---|---|---|---|
| s) | I was satisfied with my partnership | ☐ | ☐ | ☐ | ☐ | ☐ |
| t) | I had someone with whom I could talk in confidence. | ☐ | ☐ | ☐ | ☐ | ☐ |
| u) | I quickly got into quarrels and arguments | ☐ | ☐ | ☐ | ☐ | ☐ |
| v) | I was fully operational in my job/household | ☐ | ☐ | ☐ | ☐ | ☐ |
| w) | I was able to complete the most important tasks at hand | ☐ | ☐ | ☐ | ☐ | ☐ |
| x) | the care of my sick child has put a lot of strain on me | ☐ | ☐ | ☐ | ☐ | ☐ |
| y) | I had enough free time for myself | ☐ | ☐ | ☐ | ☐ | ☐ |

| | | Never | Rarely | Sometimes | Often | Always |
|---|---|---|---|---|---|---|
| z) | I had enough time for my partner | ☐ | ☐ | ☐ | ☐ | ☐ |
| aa) | I felt burdened by my child's illness | ☐ | ☐ | ☐ | ☐ | ☐ |
| bb) | we could support each other in the family | ☐ | ☐ | ☐ | ☐ | ☐ |
| cc) | we could talk openly with each other in the family | ☐ | ☐ | ☐ | ☐ | ☐ |
